# Penta-C_20_: A Superhard Direct Band Gap Carbon Allotrope Composed of Carbon Pentagon

**DOI:** 10.3390/ma13081926

**Published:** 2020-04-19

**Authors:** Wei Zhang, Changchun Chai, Qingyang Fan, Yanxing Song, Yintang Yang

**Affiliations:** 1 Key Laboratory of Ministry of Education for Wide Band-Gap Semiconductor Materials and Devices, School of Microelectronics, Xidian University, Xi’an 710071, China; ccchai@mail.xidian.edu.cn (C.C.); syx739686768@163.com (Y.S.); ytyang@xidian.edu.cn (Y.Y.); 2 College of Information and Control Engineering, Xi’an University of Architecture and Technology, Xi’an 710055, China

**Keywords:** carbon allotrope, superhard materials, direct band gap, mechanical property, stability

## Abstract

A metastable *sp*^3^-bonded carbon allotrope, Penta-C_20_, consisting entirely of carbon pentagons linked through bridge-like bonds, was proposed and studied in this work for the first time. Its structure, stability, and electronic and mechanical properties were investigated based on first-principles calculations. Penta-C_20_ is thermodynamically and mechanically stable, with equilibrium total energy of 0.718 and 0.184 eV/atom lower than those of the synthesized T-carbon and supercubane, respectively. Penta-C_20_ can also maintain dynamic stability under a high pressure of 100 GPa. Ab initio molecular dynamics (AIMD) simulations indicates that this new carbon allotrope can maintain thermal stability at 800 K. Its Young’s modulus exhibits mechanical anisotropy. The calculated ideal tensile and shear strengths confirmed that Penta-C_20_ is a superhard material with a promising application prospect. Furthermore, Penta-C_20_ is a direct band gap carbon based semiconducting material with band gap of 2.89 eV.

## 1. Introduction

Carbon science is one of the most dynamic and competitive research fields. The rapid development of carbon science has extensively and deeply affected many disciplines and various fields of high technology. Carbon science also shows strong vitality as an interdisciplinary field and has formed a research field with a wide range of subject contents and potential application prospects. Carbon atoms have *sp*, *sp*^2^, and *sp*^3^ hybridization, which enables carbon to form a variety of structures and to exist in nature in the forms of diamond, graphite, graphene, fullerenes, and nanotubes among others. In 1985, Kroto et al. [1] succeeded in synthesizing the buckminsterfullerene (C_60_) molecule by vaporizing graphite, which promoted a considerable amount of research on fullerenes. Soon after, carbon nanotubes were discovered by Iijima [2], pushing the study of carbon science to the world of one-dimensional materials. Currently, the recent discovery of layered carbon material (including graphdiyne [3], graphyne [4], and graphene [5]) is promoting carbon science to a two-dimensional (2D) field. The unique structures give fullerenes, graphene, and nanotubes a wide application prospect in nanoelectronics, biomedicine, gas detection, military defense, and so on [6,7]. To our credit, fundamental theory has improved in recent years, making it possible to predict these carbon structures. In particular, computational material science, consisting of first-principle calculations, has enabled great achievements in material design and property prediction [8,9,10,11,12,13,14,15,16,17].

To date, rich and varied kinds of carbon allotropes have been theoretically predicted or experimentally synthesized, such as C_96_ [18], carbon foam [19], *P*2/*m* C_54_ [20], M-carbon [21], W-carbon [22], C_20_-T [23], T-carbon [24], supercubane [25], and other materials [26]. The database SACADA [27] contains about 500 examples up to May 2017. After a topology-based multiscale theoretical study, Baburin et al. [28] reported six carbon allotropes with low energy from about six hundred thousand zeolite nets. These new allotropes are superhard and transparent, and they are at most 0.12 eV/atom more energetic than diamond. In 2016, Wang et al. [23] discovered a superhard all-*sp*^3^ hybridized carbon allotrope, C_20_-T, which has large cavities and porous structure. C_20_-T is a superhard carbon allotrope with a calculated Vickers hardness of about 72.76 GPa. In 2019, Zhang et al. [29] designed two superhard three-dimensional (3D) carbon allotropes, *P*6/*mmm*-C_54_ and *Cmmm*-C_32_, which consist of *sp*^3^ hybridized carbon atom. They found that *Cmmm*-C_32_ exhibits wide direct band gap semiconducting property. Using first-principle calculations, four 3D C_60_ polymers with ordered binary-alloy-type structures were investigated by Laranjeira et al. [30] In these polymers, each molecule is in one of the two standard orientations and analogous to four kinds of ordered binary-alloy-type structures. The calculated band structures show that all four polymers exhibit metallic properties. After cold compression of C_70_ peapods, Yang et al. [31] obtained an all-*sp*^3^ hybridized carbon allotrope with monoclinic unit cell, named *V*-carbon. *V*-carbon consists of five-, six-, and seven-numbered carbon rings and exhibits a honeycomb structure. The calculation shows that it is a superhard and ultraincompressible material, with a bulk modulus and hardness of 411 and 89.4 GPa, respectively. In order to predict superhard materials, Avery et al. [32] applied machine learning towards the carbon allotropes family and successfully identified 43 potential superhard carbon materials. Through topological analysis of these superhard materials, they found that phases with hardness slightly harder than the diamond phase contain a large number of diamond and/or lonsdaleite in their structures. Recently, Zhang et al. [33] proposed a cyclooctatetraene- and butadiene-based 2D fully *sp*^2^-bonded carbon allotrope, PBCF-graphene. This 2D material has a direct band gap and has higher carrier mobility than monolayer MoS_2_. Theoretical studies have shown that it can withstand equibiaxial tensile strains of 17.6% and a high temperature of 1000 K.

In this work, we obtained a new stable, semiconductor and superhard carbon allotrope Penta-C_20_, which was obtained by using the random sampling strategy combined with space group and graph theory (RG2) [34,35] to search for structures in orthorhombic systems. After analyzing its underlying topology [36], we found that it is a known net observed in the database of hypothetical zeolites collected by Deem and called PCOD8045750 [37]. Its physical properties have been systematically calculated by applying first-principle calculations. The calculated results illustrate that this novel carbon allotrope is mechanically, thermodynamically, dynamically, and thermal stable. In addition, this material exhibits a wide direct band gap.

## 2. Calculation Methods

This work was performed with first-principle calculations. Most calculations were implemented within the Vienna ab initio simulation package (VASP.5.4.4) [38,39,40], with the projector augmented wave (PAW) [41] method applied to provide pseudopotentials. The Perdew–Burke–Ernzerhof (PBE) generalized gradient approximation (GGA) [42] was employed for the exchange correlation. To expand the valance electron wavefunctions, the plane–wave cutoff energy was set as 500 eV. The Brillouin zone was sampled with 7 × 7 × 7 Monkhorst-Pack (MP) [43] special k-point grids. The convergence criteria for the total energy and the atom force calculations were taken as 1 × 10^−5^ eV and 0.01 eV/Å, respectively. Phonon frequency calculations were performed in the PHONOPY code [44], with forces calculated by density functional perturbation theory (DFPT) [45], as implemented in VASP. Based on the optimized geometry of the structure obtained from the GGA-PBE functional, the hybrid HSE06 functional [46] was used for high-accuracy electronic property calculations. The elastic constants were calculated by the Cambridge Serial Total Energy Package (CASTEP) code [47] using the GGA-PBE functional.

## 3. Results and Discussion

### 3.1. Structural Properties

**Figure 1:**
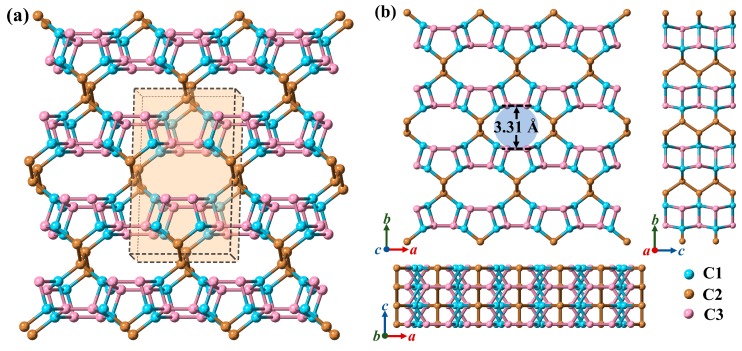
(**a**) Top and (**b**) side views of the optimized Penta-C_20_ crystal structure. The orthorhombic unit cell is shown in the light orange box in Figure 1a. C1, C2, and C3 are represented as pastel cyan, light brown, and pastel magenta spheres.

**Figure 2:**
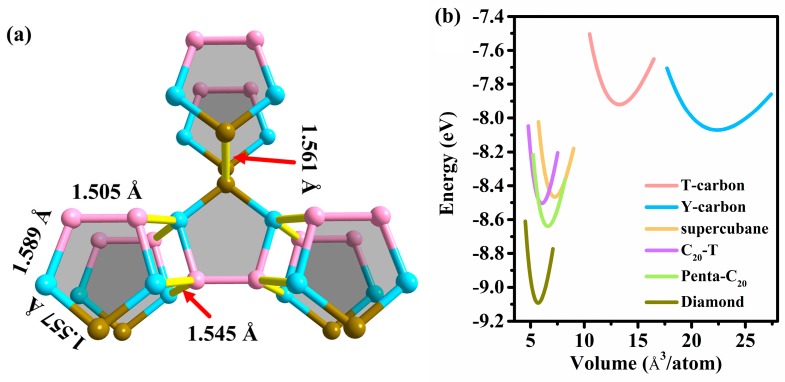
(**a**) The bond lengths and bridge-like bonds between carbon pentagons in the Penta-C_20_ structure. The bridge-like bonds are represented as bright yellow sticks. (**b**) The total energy vs the volume per atom for Penta-C_20_ in comparison with several other previous carbon allotropes.

The equilibrium crystal structure of this novel structure is depicted in Figure 1. The equilibrium structure information, including space group, lattice parameters, and density of this novel carbon allotrope are listed in Table 1, together with those of TY-carbon [48], Y-carbon, T-carbon [49], C_20_-T [23], and diamond [50] for comparison. In this work, the calculated crystal structure information of TY-carbon, C_20_-T, Y-carbon, T-carbon, and diamond are in line with previous work, proving the reliability of our theoretical work. This novel structure belongs to the orthorhombic system and has *Cmcm* (No. 63) symmetry with *a* = 5.595, *b* = 9.168 and *c* = 2.577 Å. As shown in Figure 1a, its orthorhombic unit cell consists of 20 *sp*^3^ hybridized carbon atoms. Within its unit cell, three inequivalent carbon atoms occupy the Wyckoff positions 8*g* (0.28168, 0.84609, 0.75), 8*g* (0.63477, 0.68028, 0.75), and 4*c* (0, 0.54828, 0.25), which are represented as pastel cyan (C1), light brown (C2), and pastel magenta (C3) spheres in Figure 1. This novel structure consists entirely of carbon pentagons linked through bridge-like bonds and is named Penta-C_20_, as shown in Figure 2a. In a unit cell, each C1 atom is covalently bonded with one C2 atom and three C3 atoms, while two neighboring C2 atoms and two neighboring C3 atoms are covalently bonded. Penta-C_20_ has five distinct bond lengths. Its basic carbon pentagon building block contains three distinct bond lengths, 1.557 Å (C1-C2), 1.589 Å (C1-C3) and 1.505 Å (C3-C3), while its bridge-like bonds have two different bond lengths: 1.545 Å (C1-C3) and 1.561 Å (C2-C2). The average bond length is 1.552 Å, slightly longer than that of diamond (1.54 Å). Additionally, Penta-C_20_ has eight different bond angles, namely, 89.630°, 90.370°, 103.126°, 107.077°, 110.961°, 111.360°, 112.747°, and 120.887°. Penta-C_20_ has a honeycomb structure along the *c*-axis. Its largest pore is an ellipse of twelve atoms with a short axis of 3.31 Å. In addition to the ellipse, there are also pentagons and quadrilaterals on the *c*-axis projection. The honeycomb structure causes the density of Penta-C_20_ (3.031 g/cm^3^) to be lower than the density of diamond (3.518 g/cm^3^).

### 3.2. Stability

**Figure 3:**
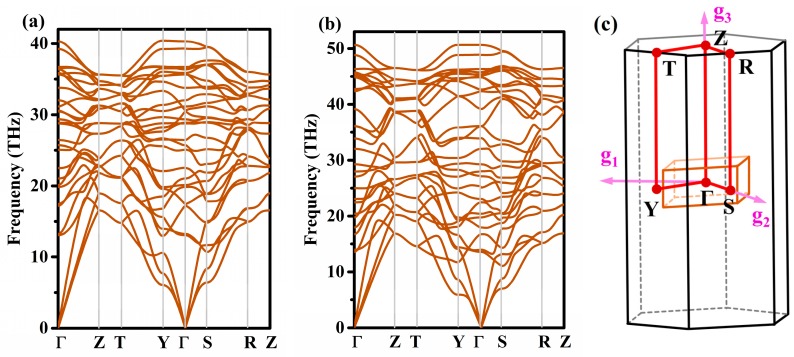
The phonon spectra of Penta-C_20_ under (**a**) 0 GPa and (**b**) 100 GPa, and the coordinates of the high symmetry points in the Brillouin zone of Penta-C_20_ (**c**).

**Figure 4:**
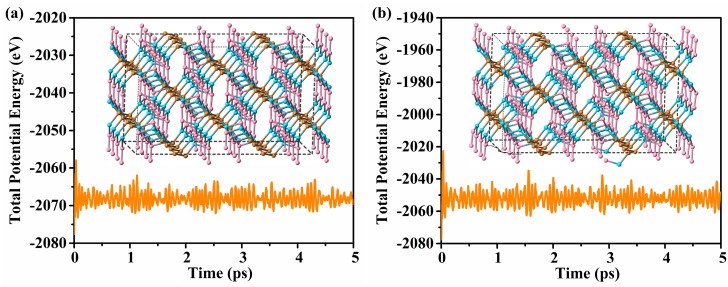
The total energy fluctuations of Penta-C_20_ as a function of the AIMD simulation at 300 K (**a**) and 800 K (**b**). The insets are the atomic configurations of 2 × 2 × 3 supercell of Penta-C_20_ at the end of the 5 *ps* AIMD simulation.

To study the thermodynamic stability of Penta-C_20_, we calculated the total energy vs the volume per atom for Penta-C_20_ in comparison with those of supercubane, C_20_-T, Y-carbon, diamond, and T-carbon, as shown in Figure 2b. In this work, the equilibrium total energies per atom of Penta-C_20_, Y-carbon, supercubane, C_20_-T, T-carbon and diamond are −8.639, −8.071, −8.455, −8.505, −7.921, and −9.093 eV/atom, respectively. Although Penta-C_20_ is metastable with energy higher than diamond of 0.454 eV/atom, it is energetically more stable than supercubane, Y-carbon, C_20_-T, and T-carbon: The equilibrium total energy of Penta-C_20_ is 0.134, 0.568, 0.184, and 0.718 eV/atom lower than those of C_20_-T, Y-carbon, supercubane, and T-carbon, respectively. Notably, T-carbon and supercubane have been synthesized experimentally by Zhang et al. [24] and Liu et al. [25], respectively. Energetically more stable than supercubane and T-carbons, this novel carbon allotrope may be synthesized experimentally. To examine the lattice dynamic stability of Penta-C_20_, we calculated the phonon spectra for Penta-C_20_ at 0 and 100 GPa, as represented in Figure 3a,b. The first Brillouin zone of Penta-C_20_ is shown in Figure 3c with the coordinates of the high symmetry points as follows: Γ: (0, 0, 0), Z: (0, 0, 0.5), T: (0.5, 0.5, 0.5), Y: (0.5, 0.5, 0), S: (0, 0.5, 0), and R: (0, 0.5, 0.5). As shown in Figure 3a and (b), the phonon spectra at 0 and 100 GPa do not show any imaginary frequencies, indicating that Penta-C_20_ can maintain lattice dynamic stability under a high pressure of 100 GPa. Furthermore, the predicted highest vibrational frequency of Penta-C_20_ at 0 GPa is 40.40 THz, which is very similar to that of *sp*^3^-bonded diamond (40.11 THz). Ab initio molecular dynamics (AIMD) simulations is a very important method to examine the thermal stability of materials. In this work, the AIMD simulations of Penta-C_20_ were performed using canonical (NVT) ensemble in a 2 × 2 × 3 supercell. The total energy fluctuations of Penta-C_20_ during the simulation time (for 5 *ps* with a time step of 1 *fs*) under 300 and 800 K are shown in Figure 4. No dramatic change was observed in structure and the total energy is almost constant, indicating that Penta-C_20_ is thermal stable at 300 and 800 K.

### 3.3. Mechanical Properties

To characterize the mechanical properties of Penta-C_20_, we examined its single-crystal elastic constant *C*_ij_, which is listed in Table 2. For an orthorhombic structure, there are nine independent elastic constants: *C*_11_, *C*_12_, *C*_13_, *C*_22_, *C*_23_, *C*_33_, *C*_44_, *C*_55_, and *C*_66._ According to Born stability criteria [51], necessary and sufficient mechanical stability criteria are:

(1) $$\begin{array}{c} C_{11}>0,\;C_{11}C_{22}>C_{12}^{2},\\ C_{11}C_{22}C_{33}+2C_{12}C_{13}C_{23}-C_{11}C_{23}^{2}-C_{22}C_{13}^{2}-C_{33}C_{12}^{2}>0,\\ C_{44}>0,\;C_{55}>0,\;C_{66}>0. \end{array}$$

**Figure 5:**
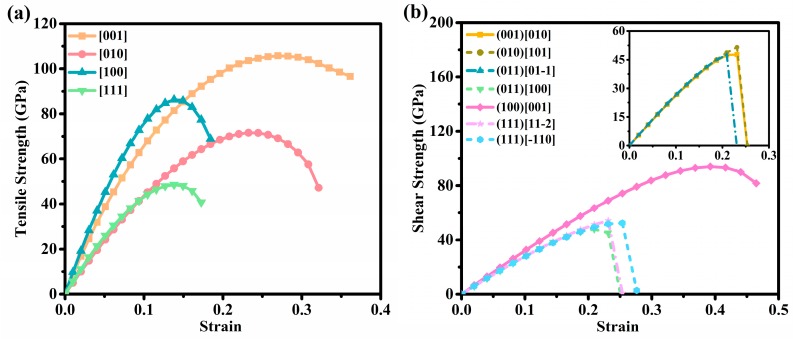
The calculated ideal tensile (**a**) and shear (**b**) strength of Penta-C_20_.

Clearly, all nine independent elastic constants of Penta-C_20_ are positive and satisfy the above criteria very well, thus proving the mechanical stability of Penta-C_20_. The elastic parameters of HS-C_48_ [52], C_96_ [18], superprismane [53], C_72_ [54], *K*_6_-carbon, T-carbon [55], and diamond [56] are also listed in Table 2. C_11_, C_22_, and C_33_ can be used to characterize the resistance of materials to elastic strain along the *x*-, *y*-, and *z*-axes. As listed in Table 2, for Penta-C_20_, *C*_11_ is higher than *C*_33_ and *C*_22_, indicating that this material is less compressible along the *x*-axis. Furthermore, C_11_, C_22_, and C_33_ values for Penta-C_20_ are higher than C_11_, C_22_, and C_33_ values for HS-C_48_, C96, superprismane, C_72_, *K*_6_-carbon, and T-carbon, as listed in Table 2. We then calculated the average bulk modulus *B* and shear modulus *G* of Penta-C_20_ according to the Voigt-Ruess-Hill approximation [60], as listed in Table 2. Penta-C_20_ has larger bulk and shear modulus (*B* = 313 GPa and *G* = 327 GPa) than HS-C_48_, C96, superprismane, C_72_, *K*_6_-carbon and T-carbon, indicating that Penta-C_20_ has stronger resistance to volume and shape changes than HS-C48, C96, superprismane, C72, K6-carbon and T-carbon. The *B*/*G* value of brittle materials is generally lower than 1.75, while that of ductile materials is greater than 1.75. [61] The *B*/*G* value of Penta-C_20_ is 0.96, indicating that Penta-C_20_ is a brittle material. This is different from C_72_, *K*_6_-carbon and T-carbon, which are ductile materials. The *B*/*G* value of Penta-C_20_ is much lower than those of HS-C_48_, C96 and superprismane, indicating that Penta-C_20_ may be more brittle than HS-C48, C96, and superprismane. Hardness is another indispensable parameter to characterize the mechanical properties of materials and has an important influence on the application of materials. In this work, we calculated the hardness of Penta-C_20_ and diamond by using the Lyakhov-Oganov model (*H_USPEX_*) [57] and Tian model (*H_Tian_*) [60]. As listed in Table 2, the hardness calculated in this work for diamond are *H_USPEX_* = 89.77 GPa and *H_Tian_* = 96.73 GPa, which are consistent with the experimental [59] and previously theoretical values. The calculated hardnesses of Penta-C_20_ are *H_USPEX_* = 76.23 Gpa and *H_Tian_* = 58.30 GPa. Considering that the hardness criterion for superhard materials is 40 GPa, the theoretical results calculated by the above two hardness models all prove that Penta-C_20_ is a potential superhard carbon material. To further determine the superhard property of Penta-C_20_, we then calculated its ideal tensile and shear strength [62], as shown in Figure 5. The figure shows that the minimum tensile and shear strengths of Penta-C_20_ reached 48.59 GP (in [111] direction) and 47.67 GP (along the (011)[0–11] slip system), respectively, which still satisfy the hardness criterion for superhard materials. Thus, it turns out that Penta-C_20_ is intrinsically a superhard carbon material.

**Figure 6:**
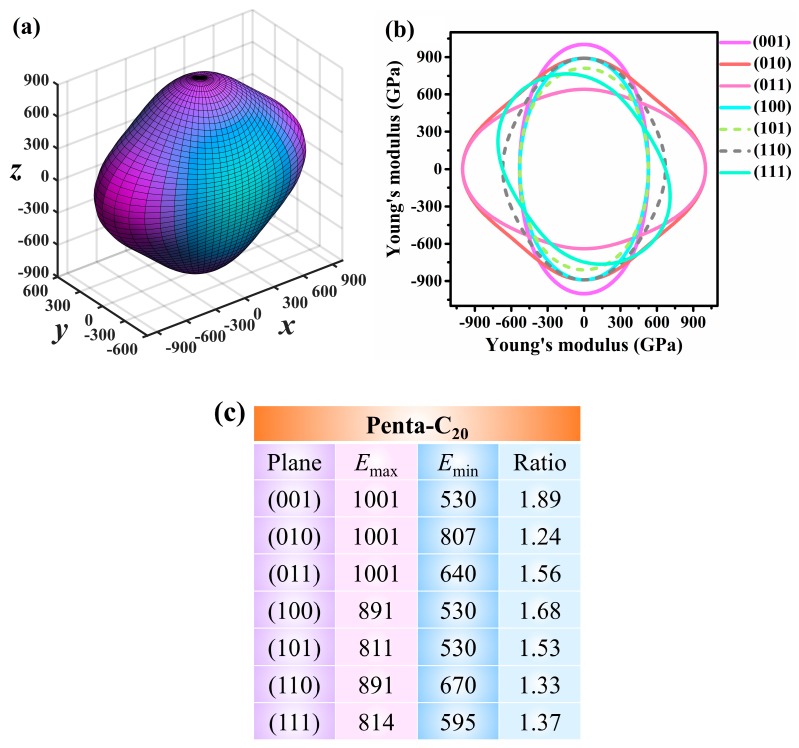
The directional dependence of the (**a**) 3D Young’s modulus and (**b**) 2D Young’s modulus for Penta-C_20_ and (**c**) the maximum Young’s modulus *E*_max_, minimum Young’s modulus *E*_min_, and *E*_max_/*E_min_* in the (001), (010), (011), (100), (101), (110), and (111) planes for Penta-C_20_.

Studying the direction dependence of the Young’s modulus is an effective and intuitive method for describing the mechanical anisotropy of materials. For the orthorhombic system, the direction dependence of the Young’s modulus can be obtained as follow [63]:

(2) $$\begin{array}{l} 1/E=S_{11}l_{1}^{4}+S_{22}l_{2}^{4}+S_{33}l_{3}^{4}+\left(S_{44}+2S_{23}\right){\left(l_{2}l_{3}\right)}^{2}\\ \;+\left(S_{55}+2S_{13}\right){\left(l_{2}l_{3}\right)}^{2}+\left(S_{66}+2S_{12}\right){\left(l_{1}l_{2}\right)}^{2} \end{array}$$

where *S*_ij_ is the elastic compliance constant; *l*_1_, *l*_2_, and *l*_3_ are the direction cosines of the direction vectors. The 3D Young’s modulus given by the above formula is illustrated in Figure 6a. The distorted 3D Young’s modulus sphere illustrates that Penta-C_20_ has inherent mechanical anisotropy. The maximum and minimum values of Young’s modulus for Penta-C_20_ are 1001 and 530 GPa, respectively, appearing on the *x*- and *y*-axes. We further show the distributions of the Young’s modulus on seven planes, namely, the (001), (011), (100), (101), (010), (110), and (111) planes, in Figure 6b. All seven main planes contain the (0, 0, 0) point. The maximum Young’s modulus *E*_max_, minimum Young’s modulus *E*_min_, and *E*_max_/*E_min_* ratio in the seven main planes are shown in Figure 6c. The distorted 2D Young’s modulus circles illustrate that Penta-C20 exhibits mechanical anisotropy in all seven planes. The maximum *E*_max_/*E*_min_ ratio reaches 1.405, whereas the minimum *E*_max_/*E*_min_ ratio is 1.24; thus, the (001) and (010) planes exhibit the largest and smallest mechanical anisotropy, respectively.

### 3.4. Electrical Properties

**Figure 7:**
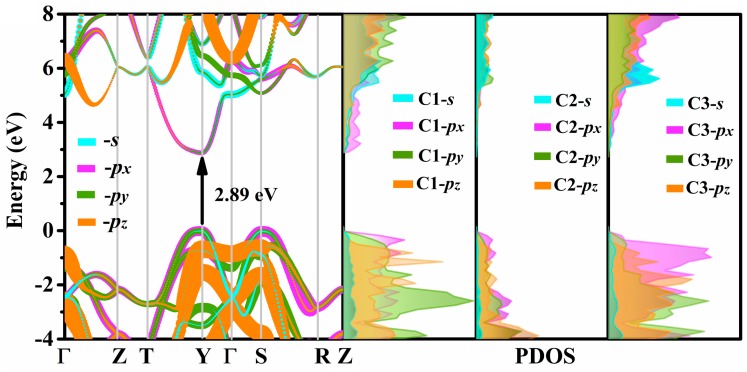
The calculated partial density of states (PDOS) and orbital projection band structure for Penta-C_20_. Cyan, magenta, olive, and orange lines correspond to the -*s*, -*p_x_*, -*p_y_*, and -*p_z_* orbitals of electrons.

The density of states (DOS) and band structure are effective methods to analyze the electrical properties of crystals. Using the HSE06 hybrid function, the orbital projection band structure and the partial DOS (PDOS) of Penta-C_20_ are calculated and shown in Figure 7. As shown in Figure 7, Penta-C_20_ is a direct band carbon based semiconducting material with band gap of 2.89 eV, as the conduction band minimum (CBM) and valence band maximum (VBM) are both found at Y point. As the orbital projection band structure shows, both VBM and CBM of Penta-C_20_ are composed mainly of the -*p_x_* orbital, although other orbital contributions are not less significant. Moreover, the electrons in the -*p_x_* orbital contribute more in VBM than in CBM. The electrons in the -*p_x_*, -*p_y_*, and -*p_z_* orbitals contribute more in the valence band than in the conduction band, while the electrons in the -*s* orbital contribute more in the conduction band. As shown in the PDOS, C2 atoms contribute the least to the orbits of the considered energy range. Both VBM and CBM of Penta-C_20_ are composed mainly of C1-*p_x_* and C3-*p_x_* orbitals.

## 4. Conclusions

Based on first-principle calculations, the physical properties (including structural properties, stability, and mechanical and electronic properties) of a novel carbon material, Penta-C_20_, were studied in detail. Penta-C_20_ can be formed from carbon pentagons linked through bridge-like bonds. In the direction of the *c*-axis, Penta-C_20_ presents a honeycomb structure composed of quadrilateral, pentagonal, and twelve-sided shapes, which makes its density lower than that of diamond. Under a high pressure of 100 GPa, Penta-C_20_ can maintain lattice dynamic stability. Penta-C_20_ is energetically more stable than supercubane and T-carbons, illustrating that it has thermodynamic stability and may be synthesized in the future. In terms of mechanical properties, Penta-C_20_ is a potential superhard carbon material and exhibits mechanical anisotropy. In terms of the Young’s modulus, the (001) plane exhibits the largest mechanical anisotropy, while the (010) plane shows the smallest mechanical anisotropy. In addition, Penta-C_20_ is a semiconducting material with a wide direct band gap of 2.89 eV. The low energy, high stability, superhard property, and wide direct band gap of Penta-C_20_ suggest that it has potential value in high-power and high-voltage devices. This work not only enriches the carbon allotrope family but also provides novel superhard direct band gap carbon materials.

## Figures and Tables

**Table 1:** The lattice parameters and mass density for Penta-C_20_ and other carbon allotropes.

Structures	Methods	Space Group	*ρ* (g/cm^3^)	*a* (Å)	*b* (Å)	*c* (Å)	*E*_tot_ (eV)
Penta-C_20_	PBE	*Cmcm*	3.031	5.595	9.168	2.577	−8.639
T-carbon	PBE	*Fd*-3*m*	1.503	7.516	-	-	−7.921
-	PBE ^[a]^	*Fd*-3*m*	1.503	7.517	-	-	−7.922
-	Exp. ^[b]^	*Fd*-3*m*	-	7.80	-	-	-
Y-carbon	PBE	*Fd*-3*m*	0.892	9.636	-	-	−8.071
-	PBE ^[c]^	*Fd*-3*m*	0.894	9.636	-	-	−8.074
TY-carbon	PBE	*Fd*-3*m*	0.524	13.459	-	-	−8.038
-	PBE ^[c]^	*Fd*-3*m*	0.523	13.460	-	-	−8.034
C_20_-T	PBE	*P*2_1_3	3.293	4.948	-	-	−8.505
-	PBE ^[d]^	*P*2_1_3	3.298	4.945	-		-
Diamond	PBE	*Fd*-3*m*	3.518	3.567	-	-	−9.093
-	Exp. ^[e]^	*Fd*-3*m*	3.516	3.567	-	-	-

^[a]^ Ref. [48]. ^[b]^ Ref. [24]. ^[c]^ Ref. [49]. ^[d]^ Ref. [23]. ^[e]^ Ref. [50].

**Table 2:** The calculated elastic constants (C_ij_, in GPa), elastic modulus (*B* and *G*, in GPa), and hardness (*H_USPEX_*, *H_Tian_* and *H_Exp_*, in GPa) for Penta-C_20_ and other carbon allotropes.

Materials	*C*_11_	*C*_12_	*C*_13_	*C*_22_	*C*_23_	*C*_33_	*C*_44_	*C*_55_	*C*_66_	*B*	*G*	*B/G*	*H_USPEX_*	*H_Tian_*	*H_Exp_*
Penta-C_20_	1020	76	97	539	59	905	289	332	299	313	327	0.96	76.23	58.30	-
HS-C_48_^[a]^	656	137	266	151	94	777	112	92	323	287	178	1.61	-	-	-
C_96_^[b]^	623	108	-	-	-	-	194	-	-	279	219	1.2	-	-	-
Superprismane ^[c]^	306	136	185	-	-	525	226	-	-	238	150	1.59	-	-	-
C_72_^[d]^	273	139	-	-	-	-	81	-	-	183	75	2.46	-	-	-
*K*_6_-carbon ^[e]^	250	187	-	-	-	-	29	-	-	209	30	6.97	-	-	-
T-carbon ^[e]^	203	136	-	-	-	-	70	-	-	159	52	3.08	-	-	-
Diamond	1053	119	-	-	-	-	566	-	-	431	524	0.82	89.77	96.73	-
Diamond ^[f]^	1076	125	-	-	-	-	577	-	-	442	634	0.83	89.72 ^[g]^	93.6 ^[h]^	96 ± 5 ^[i]^

^[a]^ Ref. [52]. ^[b]^ Ref. [18]. ^[c]^ Ref. [53]. ^[d]^ Ref. [54]. ^[e]^ Ref. [55]. ^[f]^ Ref. [56]. ^[g]^ Ref. [57]. ^[h]^ Ref. [58]. ^[i]^ Ref. [59].
